# Comparison of aortic wall quality between patients with coronary artery disease, aortic valve disease and aortic aneurysms

**DOI:** 10.1186/1749-8090-8-S1-O10

**Published:** 2013-09-11

**Authors:** J Benedik, K Pilarczyk, F Mourad, D Wendt, K Tsagakis, HA Baba, H Jakob

**Affiliations:** 1 Thoracic and Cardiovascular Surgery, University Hospital Duisburg-Essen, Essen, Germany; 2 Institute of Pathology and Pathophysiology, University of Duisburg-Essen, Essen, Germany

## Background

Currently no intraoperative test exists to predict postoperative dissection or aneurysm formation in patients operated for other reasons as aneurysms of the ascending aorta (AA). The aim of the present study was therefore to evaluate the mechanical and histological properties of the aortic wall in patients operated for coronary artery disease (CAD), aortic valve disease (stenosis (AS) or regurgitation (AR)), and for dilatation of AA of ≥ 45mm.

## Methods

Aortic walls of 396 patients (age 67.0±10.8 years) operated for CAD (n=126), AS (n=126), AR (n=57) or AA (n=87), were subjected to mechanical stress testing and postoperative histological examination. The CAD patients served as the “negative control-group”, whereas the AA patients served as the “positive control-group”. The thickness of the aortic wall and TEE parameters (aortic annulus and sinus, sino-tubular junction and diameter of AA) were compared.

## Results

The CAD group differed significantly compared to the AA group in all measured values (p<0.01), except the aortic wall thickness did not differ between the CAD and AA group. Comparing AS and AR patients presenting with CAD, (1) we observed statistically significant differences in TEE parameters and aortic wall thickness (p<0.05) and (2) we observed significant differences in the aortic wall cohesion between CAD and AI patients (p<0.05). Comparing the patients presenting with AA we observed statistically significant differences between patients with AS and AA (p<0.01). AR and AA patients differed only in TEE parameters. Furthermore, a total of 24% patients presented either with CAD or AS had a pathological cohesion test, whereas only in 18% the histological examination further confirmed these findings.

## Conclusion

AR with or without concomitant aneurysm is associated with poor aortic wall cohesion. Based on this result we recommend a more aggressive combined replacement of the ascending aorta, even in case of a borderline dilatation (45-49mm).

